# Distributed Autonomous Organization of Learning: Future Structure for Health Professions Education Institutions

**DOI:** 10.2196/28770

**Published:** 2022-01-04

**Authors:** Daniel Cabrera, Christopher P Nickson, Damian Roland, Elissa Hall, Felix Ankel

**Affiliations:** 1 Department of Emergency Medicine Mayo Clinic Rochester, MN United States; 2 Intensive Care Unit Alfred Health Monash University Melbourne Australia; 3 SAPPHIRE Group Health Sciences Leicester University Leicester United Kingdom; 4 Paediatric Emergency Medicine Leicester Academic (PEMLA) Group Children’s Emergency Department Leicester Royal Infirmary Leicester United Kingdom; 5 Office of Applied Scholarship and Education Science Mayo Clinic College of Medicine and Science Mayo Clinic Rochester, MN United States; 6 Department of Emergency Medicine Regions Hospital, HealthPartners Institute University of Minnesota Minneapolis, MN United States

**Keywords:** blockchain, multidisciplinary, credentialing, medical education, health professionals, education, decentralization, training, curriculum, instruction

## Abstract

Current health professions education (HPE) institutions are based on an assembly-line hierarchical structure. The last decade has witnessed the advent of sophisticated networks allowing the exchange of information and educational assets. Blockchain provides an ideal data management framework that can support high-order applications such as learning systems and credentialing in an open and a distributed fashion. These system management characteristics enable the creation of a distributed autonomous organization of learning (DAOL). This new type of organization allows for the creation of decentralized adaptive competency curricula, simplification of credentialing and certification, leveling of information asymmetry among educational market stakeholders, assuring alignment with societal priorities, and supporting equity and transparency.

Health professions education (HPE) institutions are hierarchical structures designed to educate and train professionals using a model of education that is chronologically sequential and geographically restricted, and resembles an assembly line. Concurrent licensure, certification, and credentialing systems are also structured in the same rigid manner [1].

The past decade has witnessed the emergence of the knowledge economy, arising from a model appropriate for the manufacturing industry and evolving toward forming information-rich, adaptive, solution-oriented, network-based systems. This new tenet is based on the paradigms of open, distributed, decentralized, and scale-free networks [2].

The advent of complex network information systems and scalable data platforms has transformed information exchange and enabled the development of sophisticated networks, where goods, financial instruments, data, and information are handled. These scaffolds also support social media and learning networks [3]. Unfortunately, HPE organizations have neither developed nor embraced these new models.

One of the fundamental technologies powering modern information exchange networks is blockchain, which can be simply described as an open market of information where the origin and flow of assets can be traced openly, securely, and trustworthily [3].

Blockchain can potentially provide a framework to support network-based knowledge management in HPE by allowing the creation, sharing, and usage of data that are distributed and stored simultaneously in warehouses open to all users. The inception, modification, and derivation of these data are possible for all members of the system, as all modifications to the system are clearly time stamped; authors are identified, and information is secured by advanced encryption. This creates a type of information that is reliable, traceable, and valid, with the ability to propagate rapidly and securely through communities of users [3]. Although blockchain appears ideal for information management, it is its ability to serve as the foundation of higher-order applications that is of paramount importance.

These blockchain-based systems enable the potential creation of the distributed autonomous organization of learning (DAOL) [4]. The DAOL constitutes a digital space where assets are negotiated autonomously and trustworthily. The DAOL can be conceptualized as a knowledge market, where goods (or digital assets such as a skill or credential) are interchanged when certain conditions are met (eg, course credit when an assessment threshold is achieved). These transactions occur automatically after prespecified conditions are met without human intermediaries or a central authority. The exchange of assets takes place using smart contracts, agreed upon by the participants of the organization before market transactions start. The contract execution is guaranteed by autonomous agents, which are algorithms that act as a digital notary for the market.

A DAOL for HPE would create a cascade of possibilities for curriculum development, licensing, certification, credentialing, and clinical practice.

First, the creation of DAOL systems will unbind disciplines (medicine, nursing, etc), institutions, locations, and time zones. Curricula will consist of a mesh of instructional modalities and microcredentialing badges creating a conceptual change from a cohorted, time-defined progression through a curricular path, leading to a progression that is nonlinear and not defined by time or location. HPE learners would be able to create adaptive learning objectives and curricula reflecting the specific knowledge and skills required for a particular job description rather than a general discipline (eg, emergency perfusionist instead of cardiac anesthesiologist specialized in extracorporeal oxygenation). This paradigm shift will likely lead to a pivot from the primacy of professional identity to a primacy of professional competency.

Second, the DAOL will allow the completion of these curricula in an automated manner once the learner has complied with the previously specified conditions (ie, smart contracts). These contracts will likely resemble entrustable professional activities mirroring clearly defined clinically based competencies. Governance of the system will rest on autonomous agents and not on human administrators or registrars, allowing faculty to focus on role modeling, coaching, assessment, and teaching clinical skills. Credentialing and licensing can be simplified, automatized, and made significantly less expensive. A DAOL system would make all necessary information open to all users; there will be no information asymmetry among players in the market.

Third, the DAOL creates a forum, through decentralized applications, for all stakeholders to participate in the design of the system. Patients, health care workers, government agencies, universities, and prospective employers would help elaborate curricula that are contextually relevant, continuously updated, and fit for purpose on communities. Existing reusable learning objects will be automatically validated, and where required, they could be created, adapted, and validated by others. At the same time, contractual conditions and requirements can be made explicit and automated, allowing for a job market that is more efficient, transparent, and equitable. This could create a learning system that reflects the diverse needs of societies throughout the world.

We believe that the DAOL constitutes a new educational exchange structure that supports the construction and validation of knowledge, and the creation of a modern learning management system. This framework allows for a new paradigm for HPE that is distributed, open, and valid, with profound implications for curriculum development, licensing, certification, credentialing, contracts, and clinical practice. The DAOL might be the answer to the calls for reimagining the future structure of HPE.

## Abbreviations

DAOL: distributed autonomous organization of learning
HPE: health professions education
